# Supplementary material for: Living on the edge: genetic structure and geographic distribution in the threatened Markham’s Storm-Petrel (Hydrobates markhami)
Source: PeerJ. 2021 Dec 24;9:e12669. doi: 10.7717/peerj.12669 (PMC8711276; doi:10.7717/peerj.12669)
Supplement: Supplemental Information 1 [file peerj-09-12669-s001.docx]

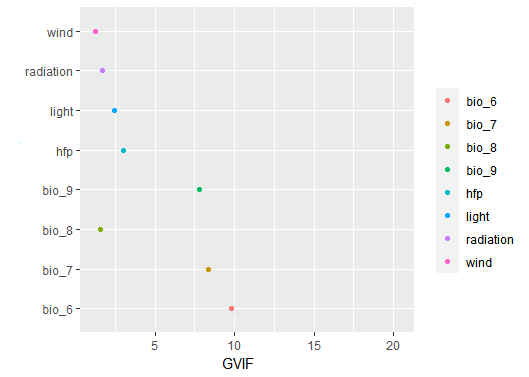


Figure S1

Plot of the variance-inflation factors (VIF) obtained for all the environmental variables after removing the bio1, bio2, bio3, bio4, bio5, bio10, bio11, bio12, bio13, bio14, bio15, bio16, bio17, bio18, bio19, elevation, topographic roughness and human population. HFP= Human footprint.


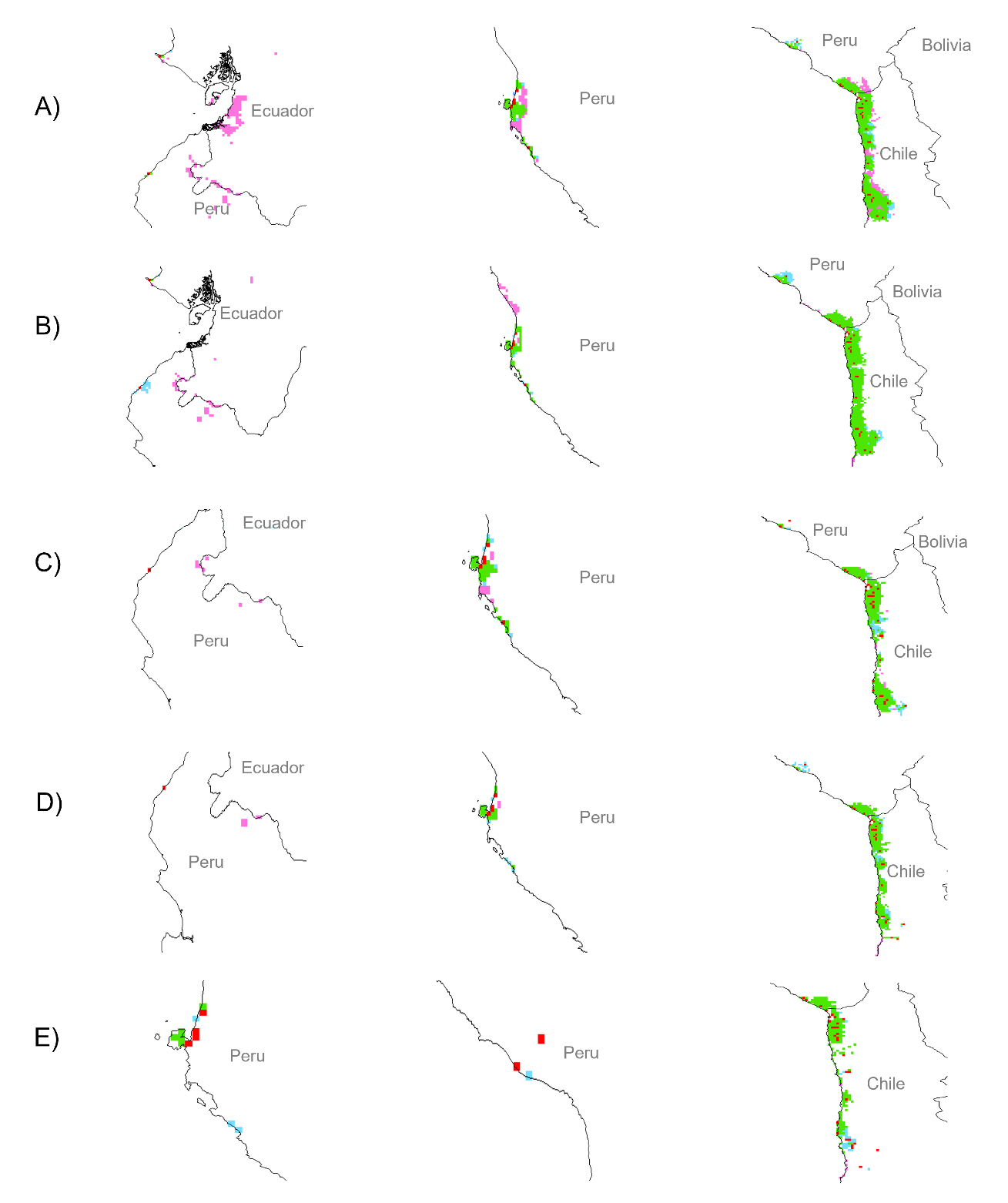


Figure S2

Dispersal restricted future distribution of *Hydrobates markhami.* Simulation for general circulation models CCMA. A) RCP 8.5, Threshold=300; B) RCP 2.6, Threshold=300; C) RCP 8.5, Threshold=500; D) RCP 2.6, Threshold=500, and E) RCP 2.6, Threshold=700. Light blue= Pixels that were once occupied but turned unsuitable after environmental change. White=Pixel that have never been occupied and are unsuitable at the end of the simulation. Red=Pixels of the species initial distribution that remained suitable throughout the simulation. Green= Pixels that have been colonized during the simulation and that remain occupied at the end of the simulation, pink=Suitable pixel that could not be colonized.


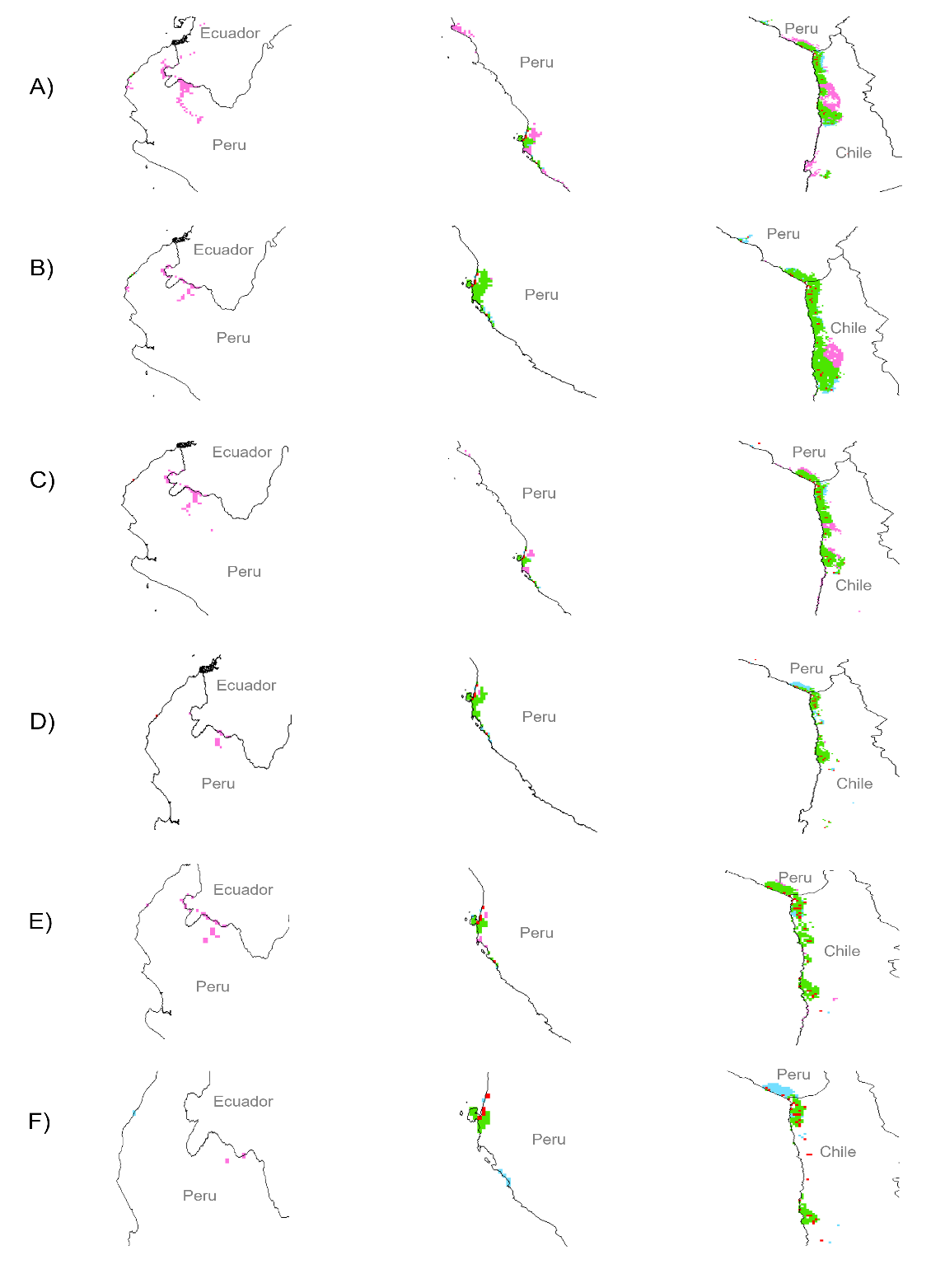


Figure S3

Simulation for general circulation model CSIRO. A) RCP 8.5, Threshold=300; B) RCP 2.6, Threshold=300; C) RCP 8.5, Threshold=500; D) RCP2.6, Threshold=500; E) RCP8.5, Threshold=700; and F) RCP 2.6, Threshold=700. Light blue= Pixels that were once occupied but turned unsuitable after environmental change. White=Pixel that have never been occupied and are unsuitable at the end of the simulation. Red=Pixels of the species initial distribution that remained suitable throughout the simulation. Green= Pixels that have been colonized during the simulation and that remain occupied at the end of the simulation, pink=Suitable pixel that could not be colonized.


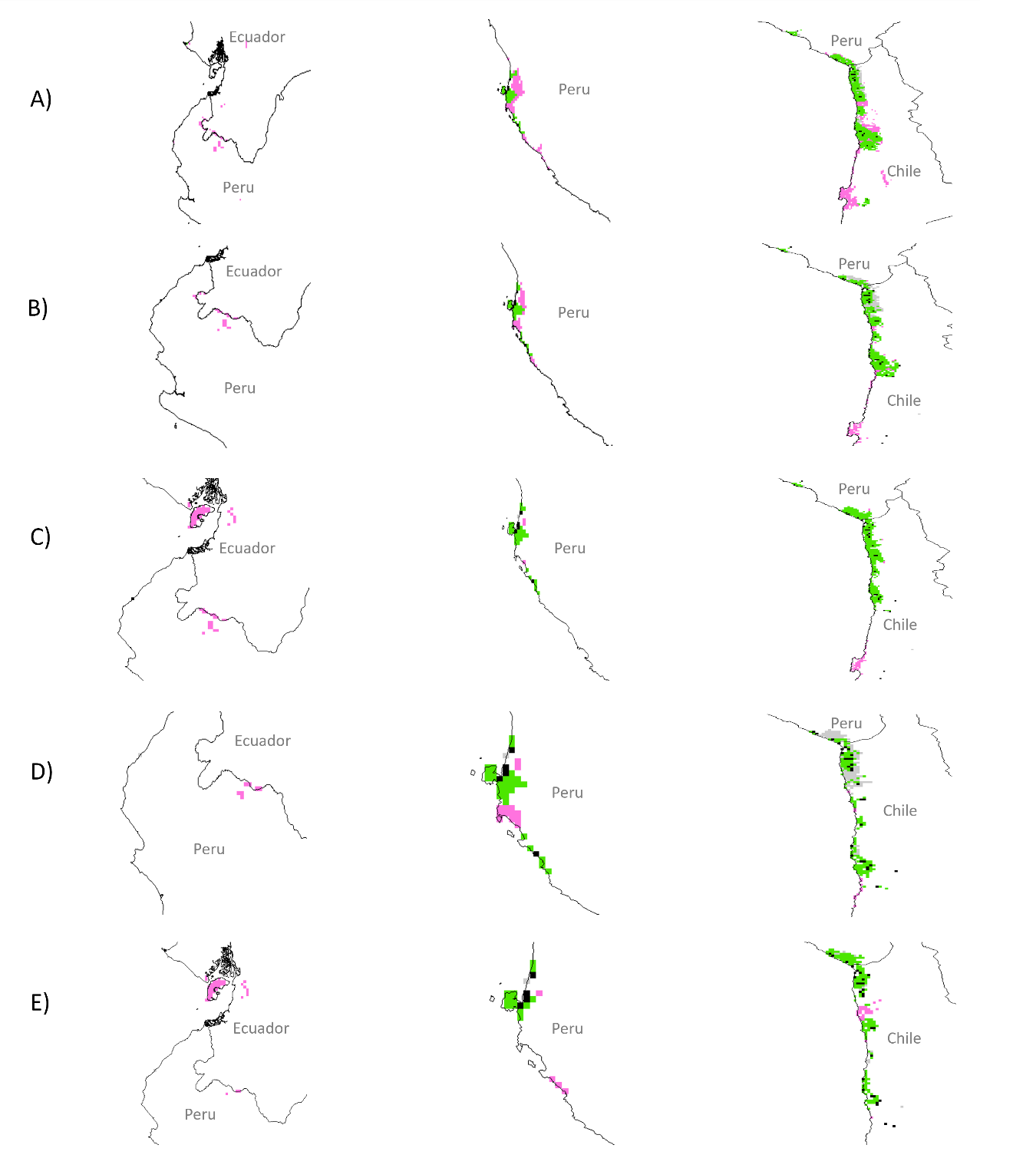


Figure S4

Simulation for general circulation model MIROC. A) RCP 8.5, Threshold=300; B) RCP 8.5, Threshold= 500; C) RCP 2.6, Threshold= 500, D) RCP 8.5, Threshold=700 and E) RCP 2.6, Threshold=700. Light blue= Pixels that were once occupied but turned unsuitable after environmental change. White=Pixel that have never been occupied and are unsuitable at the end of the simulation. Red=Pixels of the species initial distribution that remained suitable throughout the simulation. Green= Pixels that have been colonized during the simulation and that remain occupied at the end of the simulation, pink=Suitable pixel that could not be colonized.


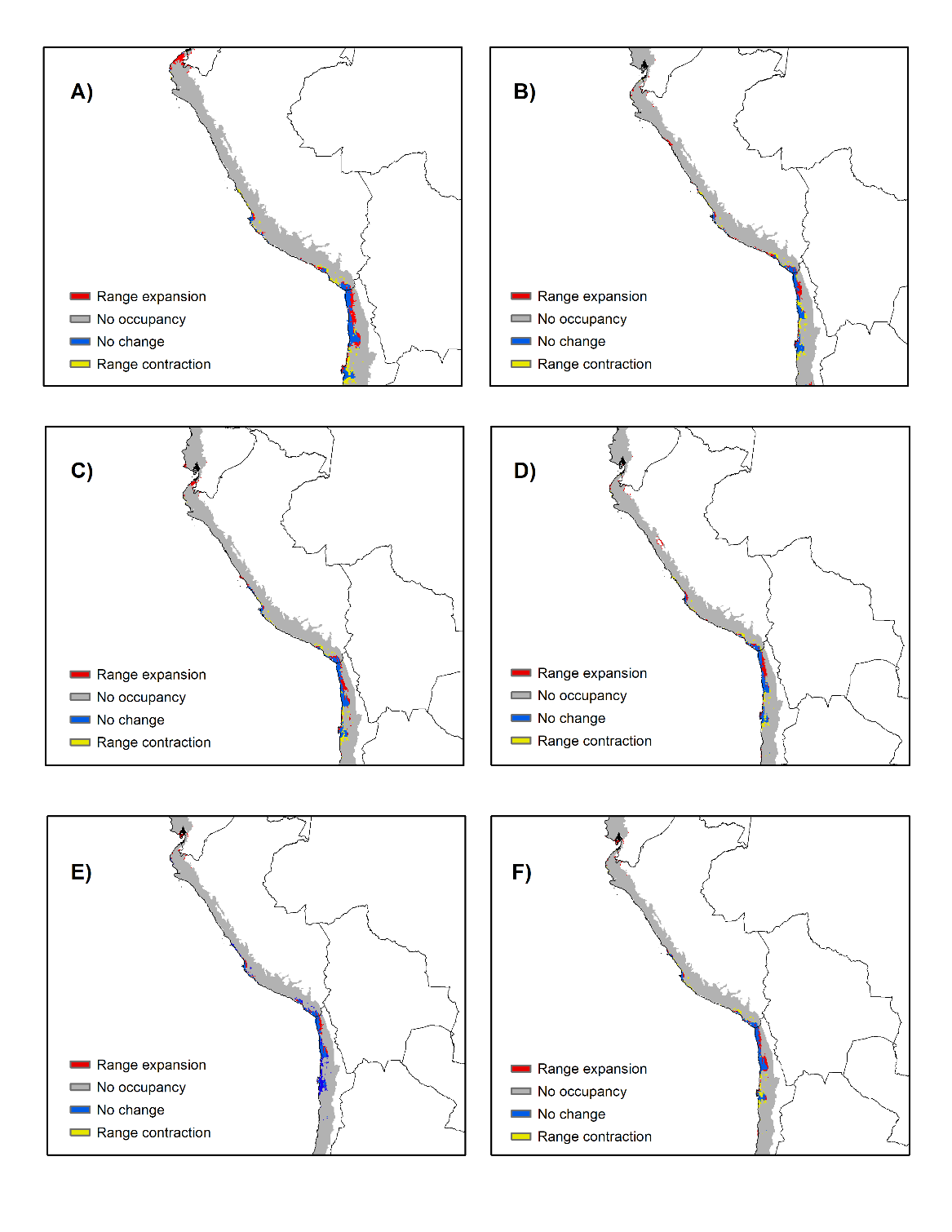


Figure S5

Distribution changes, map of expected future contraction, expansion, and areas of no change under three GCMs (CCCMA, CSIRO and MIROC) and two RCP (2.6 and 8.5). A) CCCMA (RCP 2.6), B) CCCMA (RCP 8.5), C) CSIRO (RCP 2.6), D) CSIRO (RCP 8.5), E) MIROC (RCP 2.6), and F) MIROC (8.5).
