# Supplementary material for: Living on the edge: genetic structure and geographic distribution in the threatened Markham’s Storm-Petrel (Hydrobates markhami)
Source: PeerJ. 2021 Dec 24;9:e12669. doi: 10.7717/peerj.12669 (PMC8711276; doi:10.7717/peerj.12669)
Supplement: Supplemental Information 2 [file peerj-09-12669-s002.docx]

Table S1

Taxa sample list, tissue number, country/locality and GenBank accession number.

| **N°** | **Species** | **Sample/voucher** | **Collecting locality** | **Length (bp)** | **ND1** |
| --- | --- | --- | --- | --- | --- |
| 1 | *Hydrobates markhami* | mark_317 | Peru: Cerro Lechuza, Paracas | 955 | KT186091 |
| 2 | *Hydrobates markhami* | mark_332 | Peru: Cerro Lechuza, Paracas | 955 | KT186092 |
| 3 | *Hydrobates markhami* | mark_323 | Peru: Cerro Lechuza, Paracas | 955 | KT186093 |
| 4 | *Hydrobates markhami* | mark_333 | Chile: Atacama Desert, Arica | 955 | KT186094 |
| 5 | *Hydrobates markhami* | mark_331 | Peru: Cerro Lechuza, Paracas | 955 | KT186097 |
| 6 | *Hydrobates markhami* | mark_329 | Peru: Cerro Lechuza, Paracas | 955 | KT186098 |
| 7 | *Hydrobates markhami* | Hm_01 | Chile: Pampa Chaca, Arica | 495 | MZ768852 |
| 8 | *Hydrobates markhami* | Hm_02 | Chile: Pampa Chaca, Arica | 375 | MZ768853 |
| 9 | *Hydrobates markhami* | Hm_03 | Chile: Pampa Chaca, Arica | 484 | MZ768854 |
| 10 | *Hydrobates markhami* | Hm_04 | Chile: Salar Grande, Tarapacá | 419 | MZ768855 |
| 11 | *Hydrobates markhami* | Hm_05 | Chile: Salar Grande, Tarapacá | 310 | MZ768856 |
| 12 | *Hydrobates markhami* | Hm_06 | Chile: Salar Grande, Tarapacá | 446 | MZ768857 |
| 13 | *Hydrobates markhami* | Hm_07 | Chile: Salar Grande, Tarapacá | 509 | MZ768858 |
| 14 | *Hydrobates markhami* | Hm_08 | Chile: Salar Grande, Tarapacá | 451 | MZ768859 |
| 15 | *Halocyptena microsoma* | least_804 | Mexico: Cerro Rojo, Isla San Benitos | 955 | KT186081 |
| 16 | *Halocyptena microsoma* | least_801 | Mexico: Cerro Rojo, Isla San Benitos | 955 | KT186082 |
| 17 | *Hydrobates hornbyi* | NHM-30684 | Peru | 842 | KT186100 |
| 18 | *Hydrobates homochroa* | ashy_503 | USA: Scorpion Rocks, Santa Cruz Island | 955 | KT186090 |
| 19 | *Hydrobates leucorhoa* | leach_901 | Mexico: Cerro Rojo, Isla San Benitos | 955 | KT186095 |
| 20 | *Hydrobates leucorhoa* | leach_904 | Mexico: Cerro Rojo, Isla San Benitos | 955 | KT186099 |
| 21 | *Hydrobates melania* | blk_602 | USA: Santa Barbara Island | 679 | KT186056 |
| 22 | *Hydrobates melania* | blk_654 | Mexico: Cerro Rojo, Isla San Benitos | 679 | KT186055 |
| 23 | *Hydrobates melania* | blk_603 | USA: Santa Barbara Island | 679 | KT186054 |
| 24 | *Hydrobates melania* | blk_653 | Mexico: Cerro Rojo, Isla San Benitos | 679 | KT186053 |
| 25 | *Hydrobates tethys* | otk_103 | Chile: Isla Grande, Copiapó | 955 | KT186087 |
| 26 | *Hydrobates tethys* | otk_106 | Chile: Isla Grande, Copiapó | 955 | KT186096 |
| 27 | *Hydrobates tethys* | otk_108 | Chile: Isla Grande, Copiapó | 955 | KT186089 |
| 28 | *Hydrobates tethys* | otk_109 | Chile: Isla Grande, Copiapó | 955 | KT186088 |
| 29 | *Thalassarche chrysostoma* | - | - | 1007 | AP009193 |
| 30 | *Thalassarche melanophrys* | - | New Zealand: Northland | 1007 | NC007172 |
| 31 | *Fregetta grallaria* | wbsp_711 | Chile: Isla Santa Clara, Juan Fernandez Archipielago | 955 | KT186078 |
| 32 | *Oceanites gracilis* | grac_202 | Chile: Isla Chungungo, Coquimbo | 955 | KT186069 |
| 33 | *Aphrodroma brevirostris* | - | New Zealand: Waikanae | 1007 | NC007174 |
| 34 | *Phoebastria nigripes* | - | - | 1007 | EU166974 |
