# Supplementary material for: Living on the edge: genetic structure and geographic distribution in the threatened Markham’s Storm-Petrel (Hydrobates markhami)
Source: PeerJ. 2021 Dec 24;9:e12669. doi: 10.7717/peerj.12669 (PMC8711276; doi:10.7717/peerj.12669)

Table S2

Pearson correlation matrix of environmental variables used in ecological niche modelling. Where X indicate not correlation; bio_6 = Min Temperature of Coldest Month; bio_7 = Temperature Annual Range; bio_8: Mean Temperature of Wettest Quarter; bio_9: Mean Temperature of Driest Quarter; hfp: Human footprint; light: artificial lights; radiation: solar radiation.


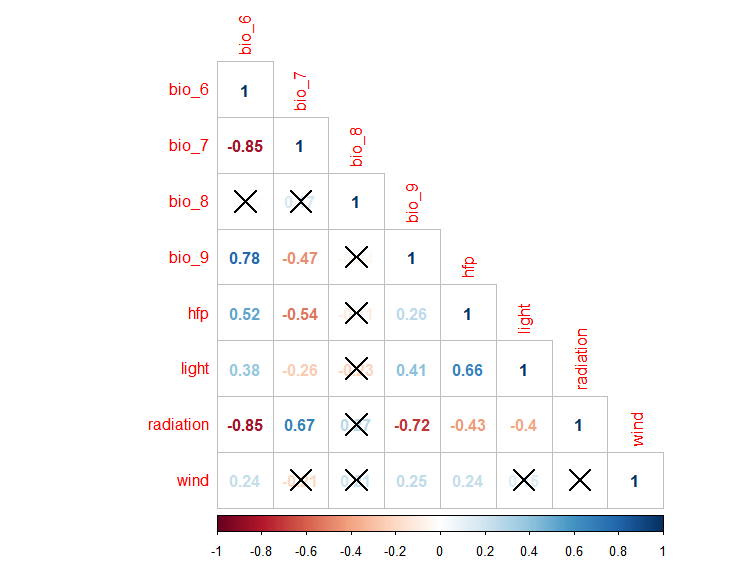

Supplement: Supplemental Information 3 [file peerj-09-12669-s003.docx]
