# Supplementary material for: Living on the edge: genetic structure and geographic distribution in the threatened Markham’s Storm-Petrel (Hydrobates markhami)
Source: PeerJ. 2021 Dec 24;9:e12669. doi: 10.7717/peerj.12669 (PMC8711276; doi:10.7717/peerj.12669)
Supplement: Supplemental Information 4 [file peerj-09-12669-s004.docx]

Table Supplementary 3.

Predicted areas (Km2) of contraction, expansion, no-change, and no-occupancy (Km^2^) for the distribution of *Hydrobates markhami*. RCP= Representative Concentration Pathway.

| **Model** | **RCP** | **Range expansion** | **No-occupancy** | **No-change** | **Range contraction** |
| --- | --- | --- | --- | --- | --- |
| CCCMA | 2.6 | 14.694 | 485.040 | 21.990 | 11.020 |
|  | 8.5 | 12.882 | 486.852 | 21.084 | 11926 |
|  |  |  |  |  |  |
| CSIRO | 2.6 | 13.385 | 486.348 | 22.896 | 10.114 |
|  | 8.5 | 12.580 | 487.153 | 22.242 | 10.769 |
|  |  |  |  |  |  |
| MIROC | 2.6 | 9.511 | 490.223 | 19.122 | 13.888 |
|  | 8.5 | 11.775 | 487.959 | 22.191 | 10.819 |
